# Commentary: Comparing caregiver burden in owners of healthy dogs with and without limb amputation: a cross-sectional analysis

**DOI:** 10.3389/fvets.2026.1885128

**Published:** 2026-07-20

**Authors:** Aneesha Verma

**Affiliations:** Jindal School of Psychology & Counselling, O.P. Jindal Global University, Sonipat, Haryana, India

**Keywords:** caregiver burden, companion animals, human dog interaction, intersubjectivity, veterinary counseling

## Introduction

Pryjmak et al. (1) compared caregiver burden between owners of dogs with limb amputation (*n* = 76) and owners of healthy control dogs (*n* = 74). Employing standardized psychological tools to measure caregiver burden and psychosocial functioning in addition to general questions on daily life, the study found no significant differences between the two groups. Caregiver demographics (age, gender, relationship status, professional background etc.) and dog-related variables (age, age at amputation, reason for amputation, affected limb etc.) revealed no effect on caregiver burden scores. However, functional changes in the dog following amputation, particularly decline in shared activities like hiking and few other social and assistive factors did influence caregiver experience.

The study makes an important contribution to veterinary literature but insufficiently explains why loss of hiking elevated caregiver burden. Although this finding was exploratory, it merits theoretical attention because it signals that burden relates to disruption of shared activities, not caring tasks alone. Disruption in established patterns of owner–dog interaction offers one plausible mechanism; others (e.g., loss of routine or identity as an “active owner”) also warrant investigation. Hiking is not merely physical exertion—it is how many owners and pets connect emotionally. Halling et al. (2) found that shared activity in natural green spaces strengthen human–dog bonds, and pet companionship literature shows such relationships enhance wellbeing (3). Loss of this connection may have psychological consequences for some owners.

Further, as the authors did not assess the quality of owner–dog interaction or owner-reported attachment to the relationship directly, it remains unclear whether changes in shared interaction contributed to caregiver burden following activity loss. The findings of the study could inform veterinary counseling more effectively if future research examined whether owner-reported attachment and patterns of reciprocal owner–dog interaction predict caregiver burden following the loss of shared activities. Activity-based interventions (finding new shared activities after limb amputation), as suggested by the study, may be of limited value if loss of hiking is not recognized as a disruption to an established pattern of reciprocal owner–dog interaction and is treated solely as a functional constraint.

## Attachment as a missing lens

The original study did not include measures of owner-reported attachment or relationship quality, nor did it examine the interactional processes through which owners and dogs negotiate changes in their relationship. Nevertheless, attachment theory provides a useful framework for understanding the owner's experience. Humans often form strong attachment bonds with their pets, and companion animals may function as primary attachment figures for their owners by fulfilling the attachment functions of proximity maintenance, separation distress, safe haven, and secure base (4) as proposed by Ainsworth et al. (5). However, as Simonen and Lohi (6) argue, understanding such relationships also requires attention to reciprocal interaction, rather than assuming direct epistemic access, or speaking on behalf of the dog's internal experience. From this perspective, disruption of shared activities following amputation may alter established patterns of interaction, potentially contributing to caregiver burden. This experience may be particularly heightened for owners who describe very strong bonds with their dogs, making changes to shared activities particularly upsetting. For example, Hawkins et al. (7) found that anxious owner–pet attachment was associated with significantly poorer owner mental health in dog owners. This suggests that owners who are more anxious about their relationship with their pet may be particularly vulnerable to psychological distress when established patterns of interaction and shared activities are disrupted. Conversely, a more secure owner attachment orientation may buffer psychological stress (7). Since the original study did not examine patterns of reciprocal owner–dog interaction, it remains unclear whether owners who experienced greater caregiver burden following the loss of hiking experienced greater disruption to established patterns of interaction.

Future research on caregiver burden in pet owners should incorporate validated owner-reported measures of attachment and relationship quality, such as the Pet Attachment Questionnaire (8), the Monash Dog Owner Relationship Scale [MDORS; (9)], and the Lexington Attachment to Pets Scale [LAPS; (10)]. These instruments provide valuable insight into owners' perceptions of their relationships with their dogs. However, they do not capture attachment as an intersubjective process, and measures grounded in reciprocal interaction are still lacking (6). Accordingly, future work should move toward conceptualizing attachment as an interactional process constituted through reciprocal interaction rather than inferred solely from owner reports. Owners reporting higher attachment anxiety, for instance, may be more sensitive to changes in their dog's activities. At the same time, differences in how owners and dogs negotiate changes in shared activities through interaction may also influence caregiver burden. Thus, caregiver burden is likely shaped not only by physical caregiving demands but also by owners' perceptions of the relationship and by changes in established patterns of reciprocal interaction. Future research should examine how owner-reported attachment orientations and interactional processes jointly influence caregiver burden following activity loss.

## Discussion

The finding that loss of hiking (and not amputation alone) predicted higher caregiver burden implies that burden arises when a shared activity that fostered emotional closeness is lost. One possible interpretation is that losing the routine of hiking interrupts the usual interactive routines between owner and dog, reducing opportunities for physical closeness and mutual comfort (4). Such interruptions may heighten owners' strain (beyond the physical tasks of care), for example, those who score high on attachment anxiety scales have shown poorer mental health when pets' routines change (7).

While the cross-sectional design cannot establish causality and the present commentary is theoretical, an empirical investigation of owner reported attachment as a mechanism is warranted. Future research should combine owner-reported relationship measures with methods that examine reciprocal interaction between owners and dogs to determine how interactional changes contribute to caregiver burden. Additionally, veterinarians should address both practical and emotional aspects of care. Veterinarians should validate the owner's grief over discontinued routines and normalize their frustration, as part of addressing the human–dog interactional changes. Suggesting alternative shared activities (e.g., gentle play, shorter walks) may help maintain the positive interaction pattern that underpins companionship (4) while maintaining opportunities for shared engagement that strengthen the human–dog bond (2). Veterinarians should recognize that ending activities like hiking can upset the usual interactional balance. Accordingly, they can support owners by acknowledging their feelings of loss and suggesting alternative ways to engage with the dog. When advising on prosthetics or physiotherapy, practitioners may benefit from framing these interventions not only as mobility aids but also as ways of supporting continued participation in shared activities that are important to the owner–dog relationship (2, 4). They should remain attentive to caregiver strain and may encourage adapted outings with carts or slings while offering empathetic guidance, which strengthens the human–animal bond (11).
